# Developing a Mobile App (iGAM) to Promote Gingival Health by Professional Monitoring of Dental Selfies: User-Centered Design Approach

**DOI:** 10.2196/19433

**Published:** 2020-08-14

**Authors:** Guy Tobias, Assaf B Spanier

**Affiliations:** 1 Department of Community Dentistry Faculty of Dental Medicine The Hebrew University - Hadassah School of Dental Medicine Jerusalem Israel; 2 Department of Software Engineering Azrieli College of Engineering Jerusalem Israel

**Keywords:** mHealth, telemedicine, public health, oral health promotion, gum health, flow of information, COVID-19

## Abstract

**Background:**

Dental visits are unpleasant; sometimes, patients only seek treatment when they are in intolerable pain. Recently, the novel coronavirus (COVID-19) pandemic has highlighted the need for remote communication when patients and dentists cannot meet in person. Gingivitis is very common and characterized by red, swollen, bleeding gums. Gingivitis heals within 10 days of professional care and with daily, thorough oral hygiene practices. If left untreated, however, its progress may lead to teeth becoming mobile or lost. Of the many medical apps currently available, none monitor gingivitis.

**Objective:**

This study aimed to present a characterization and development model of a mobile health (mHealth) app called iGAM, which focuses on periodontal health and improves the information flow between dentists and patients.

**Methods:**

A focus group discussed the potential of an app to monitor gingivitis, and 3 semistructured in-depth interviews were conducted on the use of apps for monitoring gum infections. We used a qualitative design process based on the Agile approach, which incorporated the following 5 steps: (1) user story, (2) use cases, (3) functional requirements, (4) nonfunctional requirements, and (5) Agile software development cycles. In a pilot study with 18 participants aged 18-45 years and with different levels of health literacy, participants were given a toothbrush, toothpaste, mouthwash, toothpicks, and dental floss. After installing iGAM, they were asked to photograph their gums weekly for 4 weeks.

**Results:**

All participants in the focus group believed in the potential of a mobile app to monitor gingivitis and reduce its severity. Concerns about security and privacy issues were discussed. From the interviews, 2 themes were derived: (1) “what's in it for me?” and (2) the need for a take-home message. The 5 cycles of development highlighted the importance of communication between dentists, app developers, and the pilot group. Qualitative analysis of the data from the pilot study showed difficulty with: (1) the camera, which was alleviated with the provision of mouth openers, and (2) the operation of the phone, which was alleviated by changing the app to be fully automated, with a weekly reminder and an instructions document. Final interviews showed satisfaction.

**Conclusions:**

iGAM is the first mHealth app for monitoring gingivitis using self-photography. iGAM facilitates the information flow between dentists and patients between checkups and may be useful when face-to-face consultations are not possible (such as during the COVID-19 pandemic).

## Introduction

### Barriers to Dental Care

For most people, dental visits are unpleasant, take time, cost money, and are often accompanied by discomfort or pain [1,2]. In addition, the introduction of sharp instruments and splashing water in the mouth, the need to keep the mouth open for long periods, and the sounds and smells affiliated with dental procedures further intensify the negative experience [3]. Therefore, it is not surprising that many people only seek dental care when their pain becomes unbearable and they are out of options [4].

At the time of writing, we are in the midst of an urgent public health crisis. The coronavirus disease 2019 (COVID-19) global pandemic has necessitated social distancing [5]. One field that has been affected in particular is dentistry. In Israel, the Ministry of Health [6] has prohibited nonemergency dental treatment; as such, regular checkups and elective treatments have been postponed, and dentists cannot monitor their patients’ oral health.

### Dental Scientific Background

The 2 most prevalent diseases of the oral cavity in adults are caries and periodontal diseases. Caries involves the hard tissues in the mouth (the teeth); it is caused by bacteria that initially demineralizes tooth enamel and then penetrates and causes decay [7]. Periodontal diseases involve the tissues supporting the teeth, which include the alveolar bone, periodontal ligaments, cementum, and gingivae [8]. Periodontal diseases are divided according to severity, namely gingivitis and periodontitis. Gingivitis is a reversible condition; the lesions are restricted to the gums, which are typically red, swollen, and bleeding. Gingivitis heals within 10 days of professional care and with daily, thorough oral hygiene practices [9,10]. Untreated gingivitis, however, develops into periodontitis, the irreversible stage of periodontal disease, in which bacterial toxins and the immune response to them destroy the tissues that support the teeth. As the disease progresses, the teeth may eventually become mobile or lost [11]. A significant difference between caries and periodontal disease is that caries often causes pain in its early stages, whereas periodontal diseases usually remains asymptomatic until advanced disease is noted, and sometimes there is no pain at all [12]. These two diseases share the feature of being progressive, such that a delay in treatment may lead to the need for complex and expensive treatments or tooth loss [13,14].

Most people can recognize the early signs of gingival inflammation, which are bleeding while brushing or eating something hard, an unpleasant odor, or swollen gums. But because gingivitis is not painful, people tend to postpone dental appointments to when the disease is more advanced [15]. Epidemiologically, gingivitis is common among 18-year-olds, and over 80% of the global population suffers from gingivitis from time to time [16,17]. The treatment for gingivitis is relatively straightforward, primarily based on adequate oral hygiene practices that include twice-daily brushing and interproximal cleaning using dental floss, toothpicks, and mouthwash [18,19]. In the dental clinic, gingivitis is usually treated in a single cleaning session where plaque and calculus are removed [20]. Without appropriate oral hygiene, gingivitis may return and progress into periodontitis. Treatment for periodontitis is complex and requires multiple dental appointments, and sometimes surgical intervention. During routine dental checkups, the dentist surveys periodontal health and suggests the timing of the next appointment, often in 6 months’ time. The interval between treatments is typically individualized according to variability in health behavior, lifestyle, and genetic diversity. In order to determine the optimal time interval between dental appointments, dental health should be monitored between them so that problems are detected early [21,22].

Regular checkups are essential for good oral health; however, the vast majority of people do not come for routine checkups [23]. Only 9% of people attend without an acute problem [24]. The results from a large cohort (n=608) telephone survey conducted by Sharabany et al [23] showed that 47.2% of respondents had a dental examination at least once a year, and the remaining individuals were examined once every two years or less frequently. They noted that the decision to schedule a routine dental examination was associated with socioeconomic status and personal beliefs about dental health.

### Cellular Apps Background

Approximately 45% of people have at least one of the following devices: a desktop or laptop computer, a tablet, or a smartphone [25]. In the US, 56% of citizens own a smartphone capable of connecting to the internet and downloading content [26]. About a quarter of children use their smartphones more than 5 hours a day, and about 46% of teens aged 12-17 years surf the internet for 4 hours a day (browsing from their smartphones or computers) [27].

The Apple Store and Google Play app store contain more than 318,000 medical apps, with hundreds of new apps added daily [28,29]. EHealth (electronic health) is a broad term that describes the use of electronic devices to improve health. Mobile health (mHealth) is the part of eHealth that includes the use of mobile devices to gather data about an individual's health status and provides information to professionals and patients in real-time. There are many mHealth apps, such as those that monitor medical conditions like blood glucose levels [30] (eg, SuCare, Sanofi-Aventis US LLC), blood pressure, and cholesterol [31] (eg, Vitadoc+, Medisana GmbH ). In an app for type 1 diabetes, patients can submit information and get management instructions in real-time [32] (Gluci-Chek, Roche Diabetes Care Inc). There are also apps that provide exercise programs [33] (eg, Runkeeper, ASICS), weight loss plans [34] (eg, MyFitness Pal Calorie Counter and Diet Tracker, Under Armour Inc), and functions for family planning [35] (eg, Glow, Glow Inc). Some apps are able to alert a patient to when they need further professional assistance. Other apps assist in the diagnosis of specific pathologies, such as an app that uses the image processing of urine tests to diagnose kidney disease [36] (Healthy.io). In dentistry, there are apps that show clinical procedures using 3D imagery [37] (eg, Lexi-DENTAL COMPLETE, Lexicomp). However, following an extensive search, we did not find any apps that monitor gingivitis.

To the best of our knowledge, there are no available mHealth apps that monitor the periodontal status of patients and update the dentist or the patient about dental health during the interval between visits. The oral cavity remains unmonitored, and without proper oral hygiene, gingivitis can progress and worsen.

### Purpose of the Study

The rationale for conducting this study was the understanding that between dental checkups, oral health in general, and periodontal health in particular, may decline; having an mHealth app that allows a dentist to monitor the periodontal status of patients with patient-generated photographs of the oral cavity (which we will refer to as dental selfies) should limit the deterioration that may occur between visits. Furthermore, a patient can be asked to come into the clinic when it is deemed necessary.

### Study Objective

The aim of this study was to present an mHealth app to improve the flow of information between dentists and their patients during the intervals between checkups. We describe the characterization and development of an mHealth app called iGAM [38], which focuses on periodontal health. iGAM was developed as part of a quantitative and qualitative integrated research project to promote oral health with expert writing applications. iGAM can be downloaded from both the Apple Store and Google Play store.

## Methods

### Ethics Approval and Consent

The study was approved by the Hadassah research ethics committee (IRB, 0212-18-HMO), and informed consent was obtained from all participants.

### Design and Development

The Agile approach [39] was used to develop iGAM and included the following steps: (1) user story, (2) use cases, (3) functional requirements, (4) nonfunctional requirements, and (5) the Agile software development life cycle.

The user story pertains to the conceptual scenarios of using a high-level app, aimed at defining the target audience and what is needed for the app to be self-contained for oral photography. Use cases are the specific actions that users (patients and dentists) will perform with the app. Functional requirements describe the functionality of the product, namely, which software tasks it must perform, the scope of the system, the boundaries of the product, and relationships to adjacent systems. Nonfunctional requirements describe the look and feel of the system, such as the visual characteristics [eg, the user interface (UI) and user experience (UX) of the system], its usability, and performance requirements (eg, how big, how fast). The Agile software development life cycle involves dividing the project into small increments to allow rapid changes to be made. The development life cycle includes customer satisfaction, delivering working software frequently (weeks rather than months), building around motivated individuals, using working software as the primary measure of progress, continuous attention to technical excellence and good design, and the maintenance of simplicity.

## Results

### User Story

The routine activity of 2 dental clinics, over the span of two 9-hour workdays, was monitored by the lead researcher (a dentist) and the app developer. Communication and patient management were observed in order to identify the digital dental technologies used and to assess the need for the iGAM app. We observed that dentists frequently employ administrative, communicative, clinical, and diagnostic technologies.

Then a focus group meeting was held with 10 participants: 2 dentists, 2 advanced-year dental students, and 6 patients who had just been treated. During the meeting, several questions were asked by the lead investigator using a guide. The conversation was recorded and transcribed with the prior approval of the participants. All participants believed in the potential of a mobile app to effectively monitor gingivitis and reduce the severity of gingivitis. The meeting also covered other issues, such as having access to easily understood information on gum diseases (including pictures). Some participants voiced concerns about information security, privacy issues, and the risk of personal oral health information being leaked.

Subsequently, we conducted 3 semistructured in-depth interviews on the use of cellphone apps for monitoring gum infections in the oral cavity. The mean duration of the interviews was 50 minutes. The interviewees were 2 men and 1 woman, which included a dentist, a nurse, and a dental student after dental treatment. The interviews were recorded and transcribed with permission. From the analysis of the interviews, 2 major themes emerged: (1) “What’s in it for me?” and (2) the need for a take-home message. The first theme highlighted that users need to perceive a gain from the use of the app. For example, one participant said,

I feel good. Occasionally I see some bleeding when I brush. So what? It doesn't mean anything to me, doesn't hurt me, and I don't have time to go to a dentist. For me to use this app means there has to be a reason, and I don't understand what I will get out of it.

The second theme highlighted the user’s need for a take-home message. For example, some participants said,

I would love to get answers to questions, and the doctor does not have time to answer me properly.

I would be happy if the dentist in the clinic would give me a leaflet with an explanation of how to brush my teeth; I'm not sure I brush right.

It's important for me to know that my mouth is healthy, that I'm doing things right.

### Usage Scenarios

Once we understood what the users needed, we were able to describe the use cases of the app.

#### Architecture Diagrams

**Figure 1:**
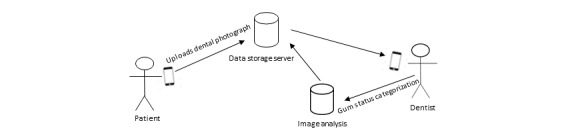
Interaction between the user, app administrator, and data storage server.

The iGAM mHealth app has 2 modes: (1) the patient mode, and (2) the dentist mode (Figure 1). In the first mode, the patient takes dental selfies (photographs of their gums) and sends them to the dentist through the app. In the second mode, the dentist determines the gingival status of the patient.

#### Use Cases: The Patient

**Figure 2:**
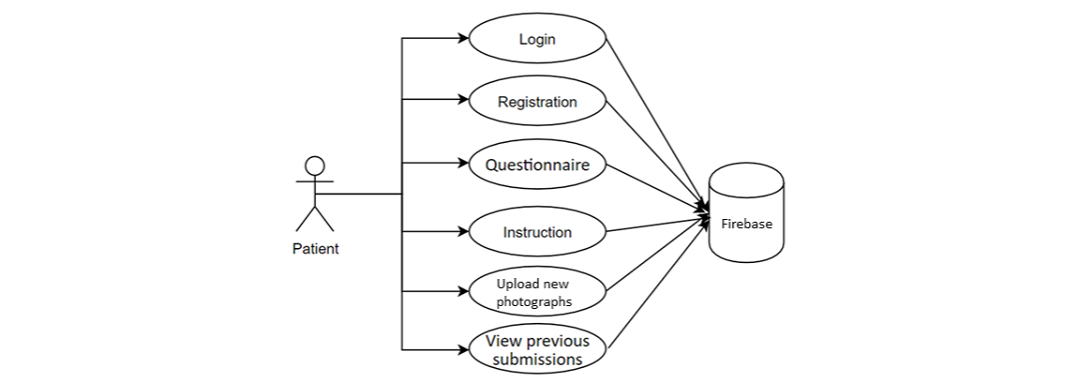
Patient use cases.

The app’s patient mode allows patients to: (1) login; (2) register and see general guidelines; (3) respond to questionnaires about health and dental behavior; (4) view tutorials with brushing instructions, information about periodontal problems, self-photography training, etc; (5) take photos; (6) and keep track of responses to previously submitted photographs (Figure 2). All submitted patient data is saved in the database. The patient is instructed to take multiple dental selfies of their gums using the rear camera of their cellphone, once a week for 8 weeks. This time period was selected based on studies on gingivitis [40].

#### Use Cases: The Dentist

**Figure 3:**
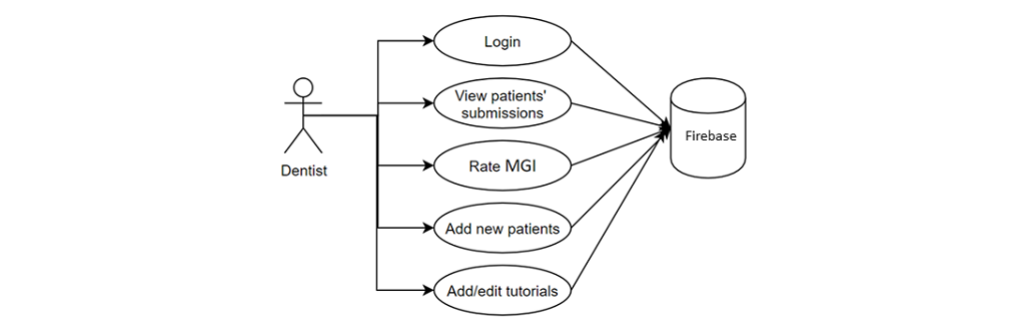
Dentist use cases.

The app’s dentist mode allows the dentist to: (1) login; (2) view the submitted dental selfies; (3) rate the image quality and evaluate gingival health status using the modified gingival index (MGI; the MGI was introduced in the mid-1980s and was found to be reliable in the visual diagnosis of gingivitis) [41]; (4) add new patients; and (5) add and edit tutorials (Figure 3).

### Functional Requirements

The following describes the intended patient user’s experience with the iGAM app.

Following initial registration, a password known only to the patient is given to maintain anonymity. The first time the patient logs in, personal patient data (such as age, gender, origin, etc) is collected. In order to characterize the patient’s interest in the app, the patient is prompted to fill out a questionnaire about dental knowledge and behaviors, including oral hygiene habits. Tutorials about oral hygiene and information on the dangers of poor oral hygiene maintenance are available on the app for the patient.

The patient is then instructed to photograph themselves once a week for 8 weeks. The dentist will assess inflammation using the MGI.

### Nonfunctional Requirements

An intuitive UI was created to allow the user to perform editing tasks by clicking twice on the main screen. Data is synchronized to the cloud after each patient session. The app opens within 2 seconds. Communication with the text-to-speech (TTS) server is established in less than 30 seconds.

**Figure 4:**
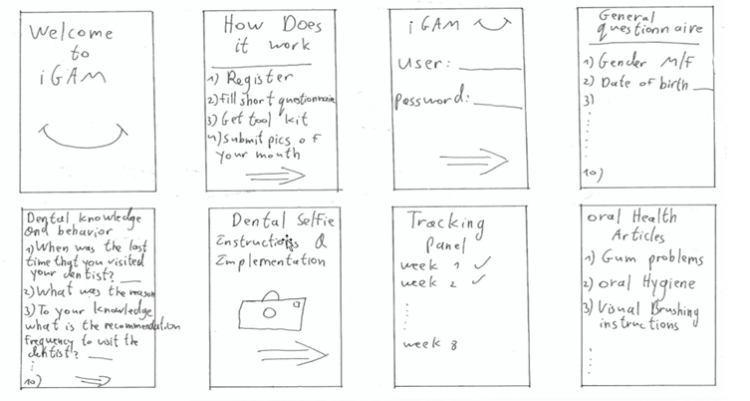
Sketches of the essence of the app.

For interface design (UI/UX), the lead researcher and app developer sketched their ideas using pencil and paper (Figure 4).

The prototype, which was designed using Adobe XD software (Adobe XD 21.0, Adobe Inc), included interface items with visual elements and colors, such as screens for the patient (Textbox 1, Figures 5-17) and screens for the dentist (Textbox 2, Figures 18-21). This facilitated the receipt of early feedback in the design process. Hebrew was selected as the interface language as it is the primary language of the users; in the future, the app will also be available in English, Arabic, and Russian, the most commonly used languages in Israel.

**Prototype screens for patients in the iGAM app.**

**Figure 5:**
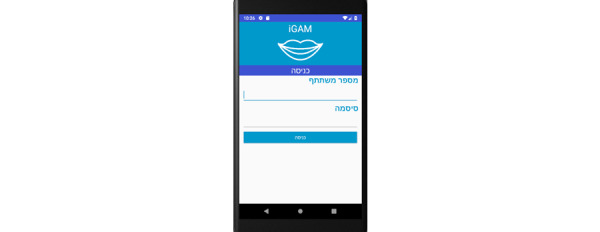
Initial login screen for the patient: patient name and password.

**Initial Login Screen** (Figure 5)

- Patient’s name and password

**Figure 6:**
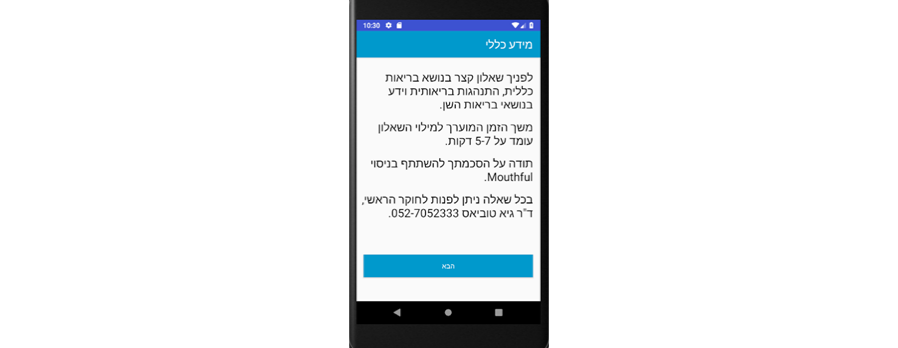
General explanation screen for the patient.

**General Explanation Screen** (Figure 6)

**Registration Questionnaire Screens**

**Figure 7:**
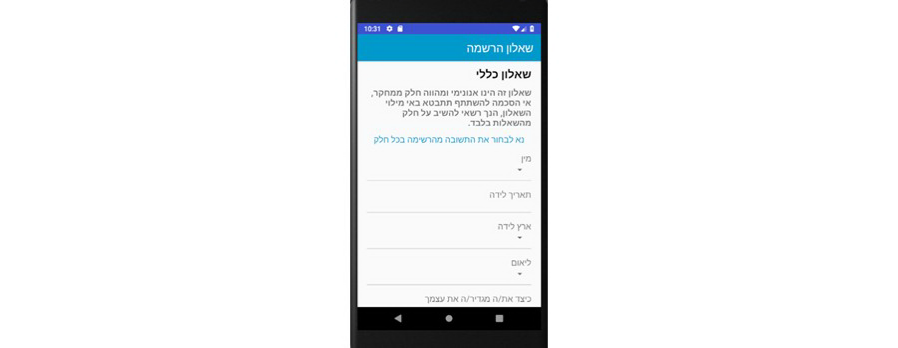
Registration questionnaire screen for the patient: general.

**Figure 8:**
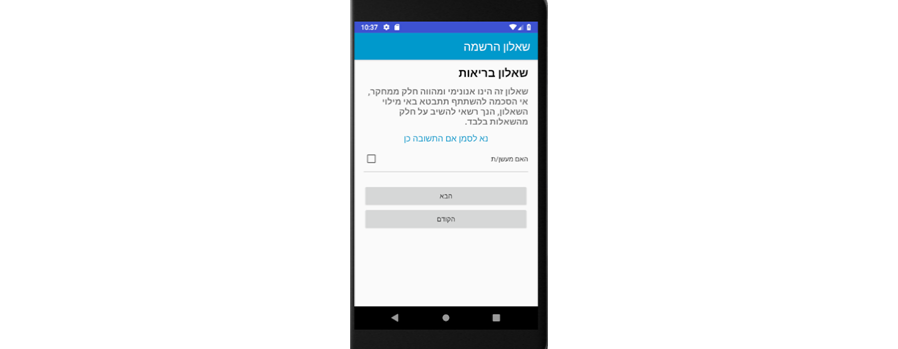
Registration questionnaire screen for the patient: general health.

**Figure 9:**
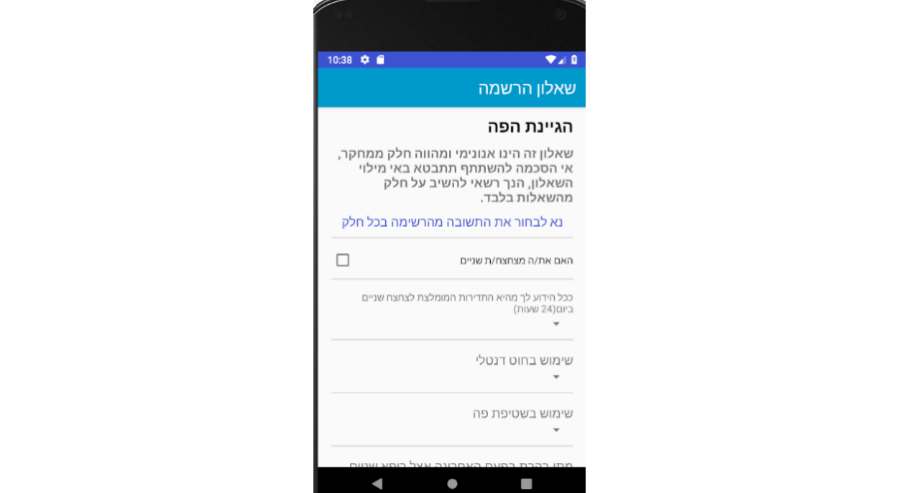
Registration questionnaire screen for the patient: oral hygiene.

**Figure 10:**
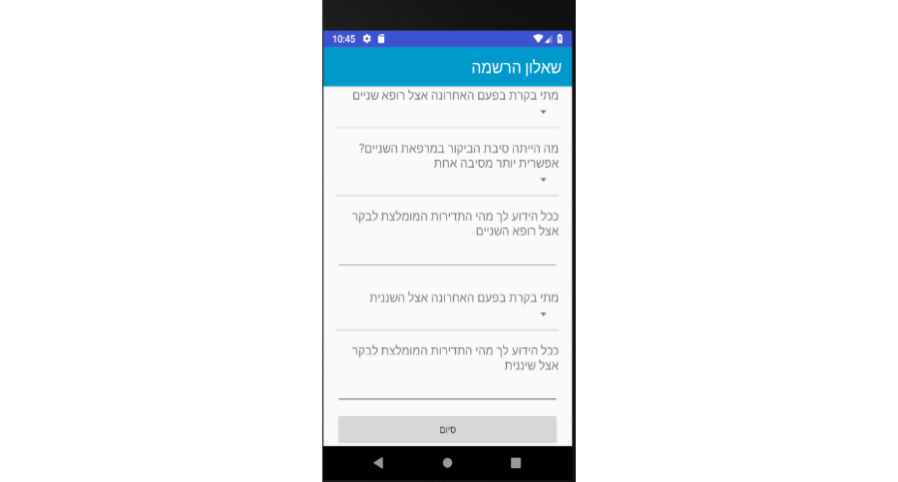
Registration questionnaire screen for the patient: oral hygiene, continued.

- General (Figure 7)
- General health (Figure 8)
- Oral hygiene (Figure 9)
- Oral hygiene, continued (Figure 10)

**Tutorial Screens**

**Figure 11:**
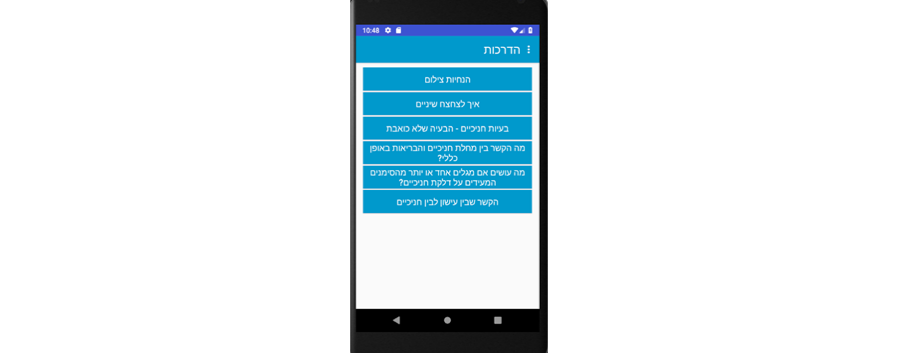
Tutorial screen for the patient: main.

**Figure 12:**
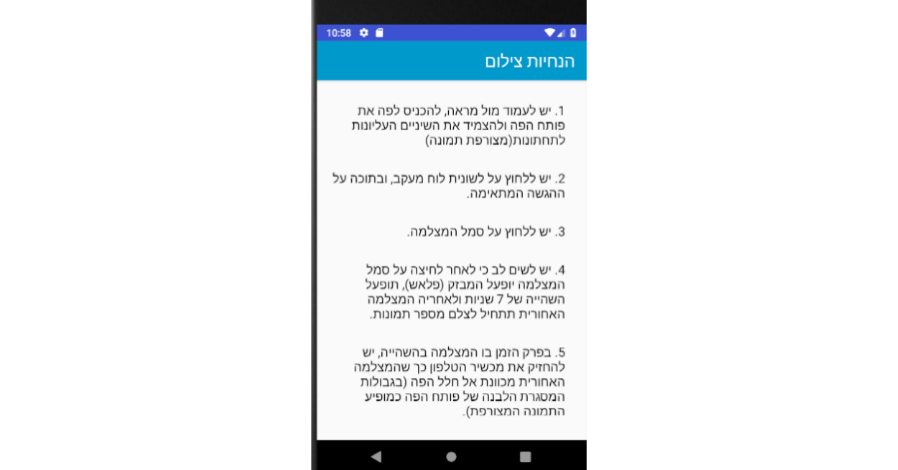
Tutorial screen for the patient: photo guidelines.

**Figure 13:**
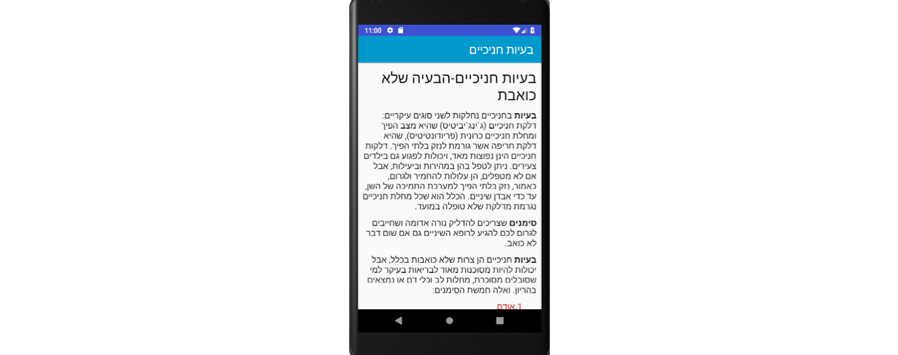
Tutorial screen for the patient: periodontal problems.

**Figure 14:**
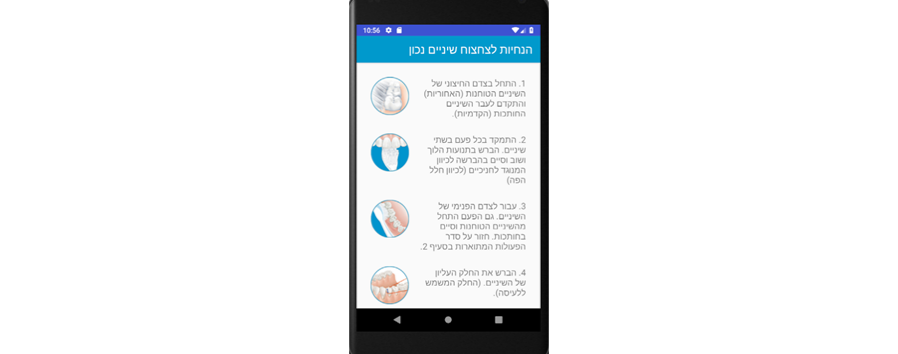
Tutorial screen for the patient: brushing your teeth.

- Main screen (Figure 11)
- Photo guidelines (Figure 12)
- Periodontal problems (Figure 13)
- Brushing your teeth (Figure 14)

**Photo Panel Screens**

**Figure 15:**
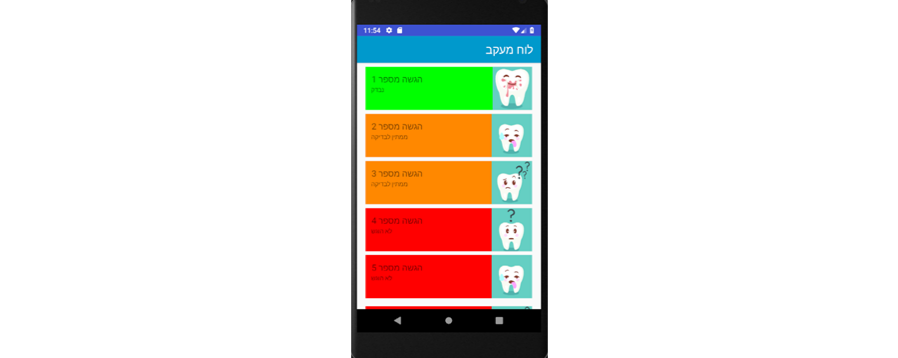
Photo panel screen for the patient: A button on each line allows a photograph to be taken at each prescribed time, and opens the camera.

**Figure 16:**
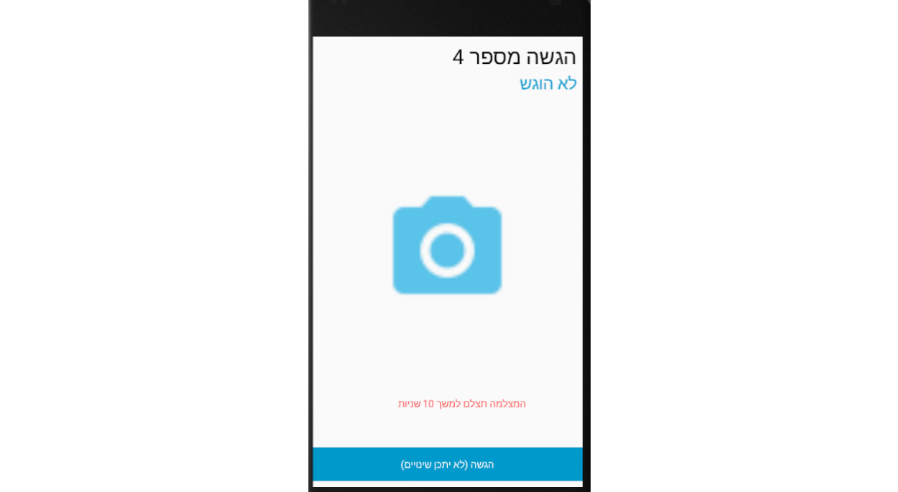
Photo panel screen for the patient: Clicking on the icon opens the camera.

**Figure 17:**
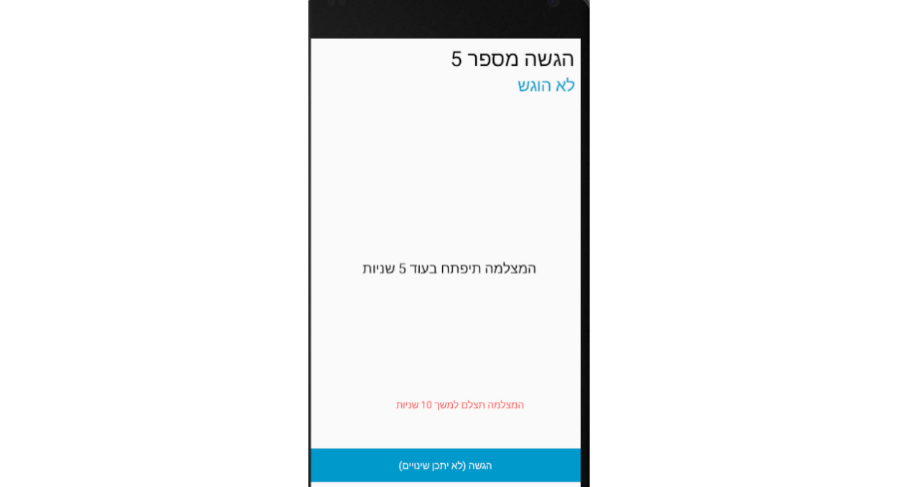
Photo panel screen for the patient: After a 10-second countdown, photographs are taken.

- A button on each line allows a photograph to be taken at each prescribed time, and opens the camera (Figure 15).
- Clicking on the icon opens the camera (Figure 16).
- After a 10-second countdown, photographs are taken (Figure 17).

**Prototype screens for dentists in the iGAM app.**

**Figure 18:**
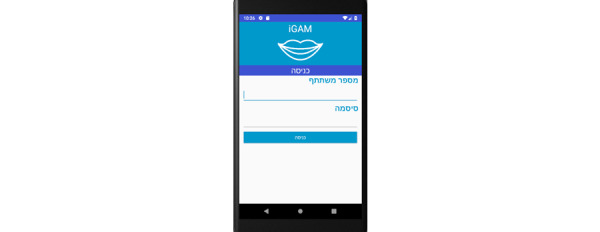
Login screen for the dentist.

**Initial Login Screen** (Figure 18)

**Figure 19:**
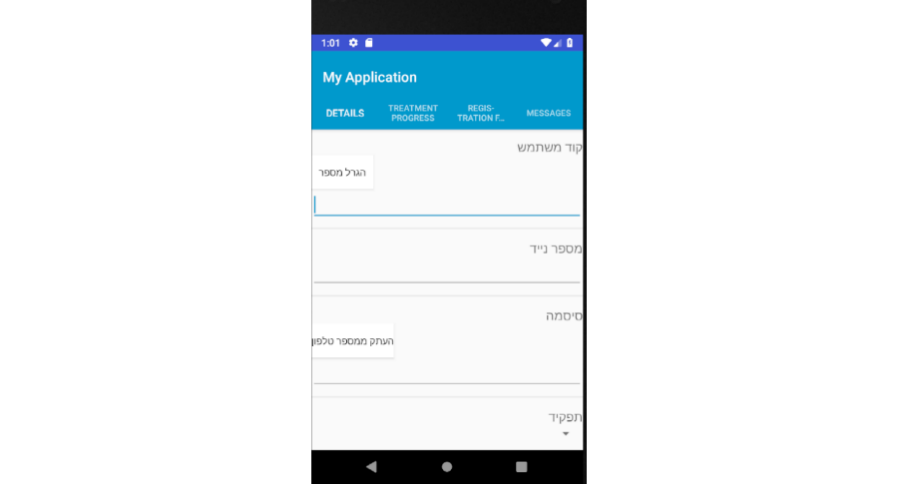
Patient screen for the dentist: the dentist may add a new patient.

**New Patient Screen** (Figure 19)

- Add a new patient

**Figure 20:**
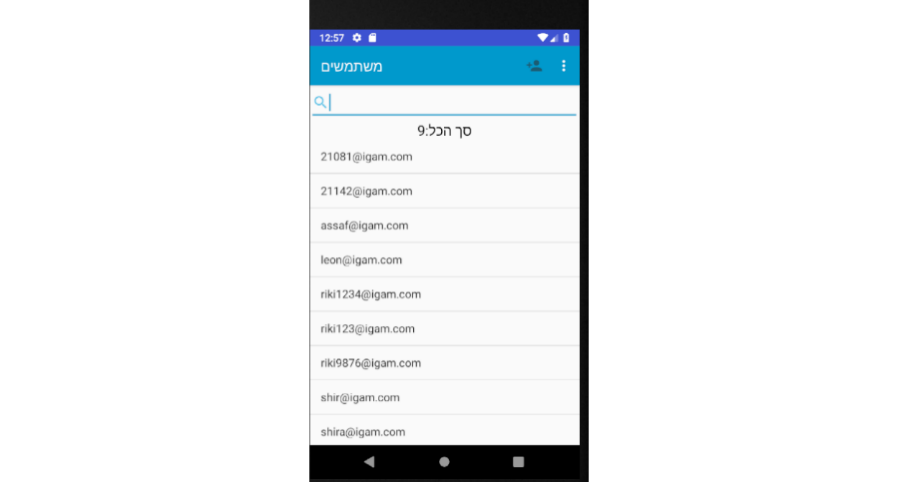
Patient list screen for the dentist: the dentist can view the list of patients.

**Patient List Screen** (Figure 20)

- The dentist can view the list of patients.

**Figure 21:**
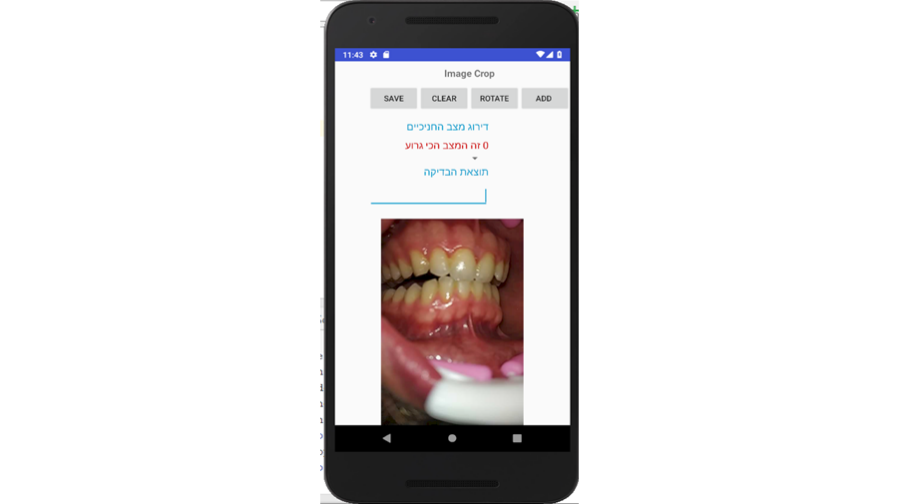
Viewing screen for the dentist: the dentist can view the dental selfie and rate the patient's condition.

**Viewing Screen** (Figure 21)

- The dentist can view the dental selfie and rate the patient's condition.

### Design and Development

**Figure 22:**
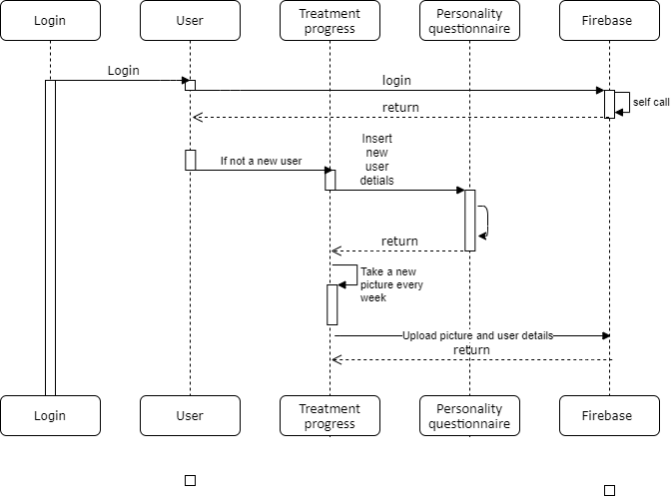
Sequence diagram of the patient’s use processes with the app; DB: database.

The sequence diagram in Figure 22 describes the processes involved in the use of the app by the patient.

After logging in for the first time, the patient fills out a form with their personal information and oral health information. The data is stored in the database (firebase) under a unique and anonymous identifier. All photographs and responses to the questionnaires are saved in the database, including the time and date of submission. After uploading a new dental selfie, the patient’s status changes to “pending review.”

**Figure 23:**
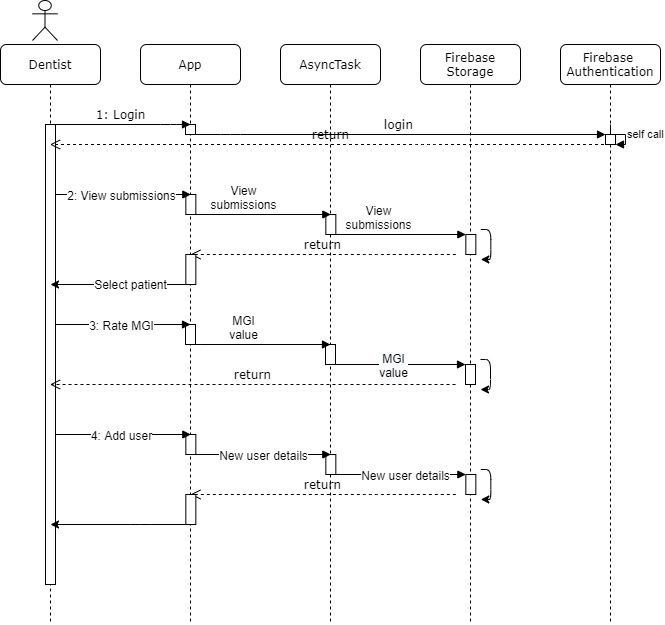
Sequence diagrams of the dentist side. After login the dentist can view patient submissions, rate gum status according to the MGI, add new patient and new tutorials.

The 4 major operations of the dentist (Figure 23) are (1) the verification of correct username and password, (2) viewing the list of new patient submissions and selecting the user to view, (3) formulating a rating using MGI, and (4) adding a new user to the trial.

### Implementation Technologies

#### Client Side

Two versions of the client side of iGAM were created. The first version was written for the Android operating system (ie, a native app, a method used for writing software in a programming language for certain types of mobile phones) in the Android Studio workspace. The second version was a hybrid app; development was done with web technologies (HTML, CSS, and mainly Javascript programming language) and was then packaged using tools like Phonegap/Cordova. We used an Ionic library to create the UI for this mobile app. The Ionic library runs on a wide range of Android iPhone devices. The technology environments we used were Android Studio for Android and Ionic Studio integrated development environment for Ionic. The development languages we used were Javascript for Android, and JavaScript, Angular, and TypeScript for Ionic.

#### Server Side

To store images and questionnaire results, Firebase storage was used. Firebase storage provides secure uploads and downloads for apps regardless of network quality, and the service is backed by Google Cloud storage.

The system data is managed by a database containing patient information. Specifically, 5 tables in the Firebase database were defined: (1) settings, (2) single shot, (3) submission reviews, (4) user labeling, and (5) user data. The settings table saves all app settings. The single-shot table saves the first image taken by each patient. The submission reviews table contains the dentist’s reviews. The user labeling and user data tables contain patient information such as answers to the registration questionnaires, the number of photographs taken, the dates on which the photographs were taken, etc.

#### Software Tests

To test the possibilities of crashes or error cases, we tested the app on a number of Android and Apple mobile phones. We confirmed the operation of (1) system activation and loading by visualizing the home screen and the loading of links; (2) the existing user authentication; (3) the opening of the camera, the saving of images, and the uploading of the images to the database; (4) the dentist ratings using MGI, and the ability to add notes; and (5) new user additions, verifications, and unique patient identification assignment.

### Agile Software Development Evaluation

The Agile software development life cycle approach was used for this research; the agile cycle involves dividing the work into small increments, allowing the product to adapt to changes quickly.

Following each photo session, several dental selfies are uploaded to the server (Firebase database). The dentist section of the app enables the dentist to view the patient’s dental selfie, to rate its image quality, and to evaluate gingival health status.

#### Agile Software Development

After initial app development, a pilot study with a group of 18 participants aged 18-45 years (the inclusion criterion was 18 years of age and above) and with different levels of health literacy and education was conducted. The participants were given a kit containing toothpaste, a toothbrush, mouth wash, toothpicks, and dental floss to ensure they had the materials needed to improve their oral hygiene. After installing the app, the participants were asked to photograph their gums once a week for 4 weeks, and then to answer 3 open-ended questions: (1) Describe the feasibility of using the app and mouth opener; (2) Describe the usability of the photo feature; (3) Describe app acceptance over the time of the study.

**Figure 24:**
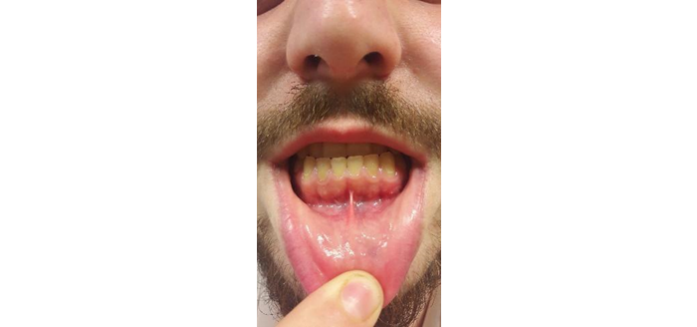
Physical positioning for taking a dental selfie: without a mouth opener (suboptimal).

**Figure 25:**
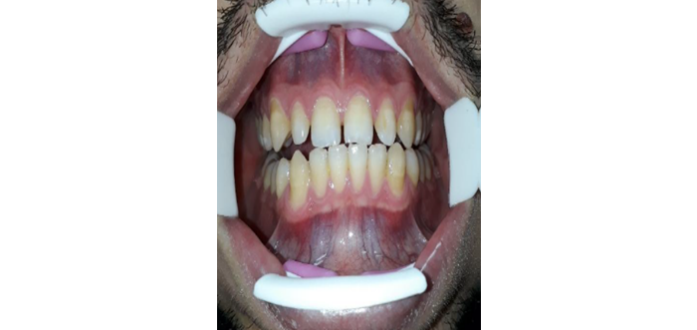
Physical positioning for taking a dental selfie: with a rigid mouth opener (suboptimal).

**Figure 26:**
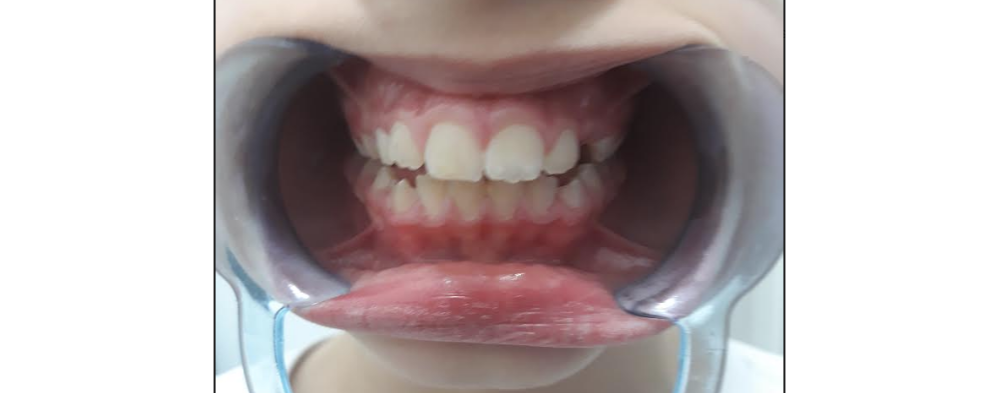
Physical positioning for taking a dental selfie: with a comfortable mouth opener (optimal).

Qualitative analysis of the answers involved 5 cycles. In cycle 1, we noted that some of the photographs were out of focus, so we reprogrammed the app to take 10 pictures each time to allow the researcher and dentist to analyze the best one. During cycle 2, 12 participants found that the physical positioning required to take the photograph (ie, holding the phone with one hand and aiming the camera at the gums with minimal movement while rolling down the lower lip with the other hand, as shown in Figure 24) to be clumsy and uncomfortable. Therefore, mouth openers (Figure 25) were added to the kit so that the user would not have to use their hand to retract their lip. The mouth opener was delivered to each participant, and as most participants found it difficult to use because of its rigidity, another, more comfortable mouth opener was tested and implemented (Figure 26), and the participants were satisfied.

In cycle 3, 5 participants reported difficulty with operating the phone, turning on the camera and the flash, and then pressing the photo button with one hand. Therefore, we reprogrammed the app so that a photo could be taken using the volume button, and the flash was set up to turn on automatically and to simultaneously initiate a 10-second timer, giving the participant time to position the camera. A sound indicated that the phone had started taking photographs, and another sound signaled the end of the photographing time.

During cycle 4, 13 participants reported difficulty remembering when to take another photo. Therefore, a weekly reminder feature (in the form of an SMS text message) was added.

In cycle 5, 8 participants reported difficultly placing the rear camera in the correct position. Therefore, users were instructed to stand in front of a mirror while taking the dental selfie, or to take pictures in the restroom behind a white wall, using natural light.

## Discussion

### Principal Findings

There are currently more than 318,000 medical apps that help diagnose and manage illness; for example, in recent years, many applications have been developed to monitor blood pressure or electrocardiography. Smartphones have very strong computing capabilities and have become involved in our daily lives [42,43]. Photography is a very valuable tool for documenting illness, supervising care, and educating patients. Most smartphones have cameras that can take and transfer high-quality dental selfies, and can also process and transmit audio and video files [44].

In this paper, we describe the characterization and development of a cellular app (iGAM) to monitor gingivitis, a prevalent condition of reversible periodontal inflammation. To the best of our knowledge, there are apps for improving oral hygiene [45,46], but no computerized platform exists to monitor gingivitis between dental appointments. This app was developed in response to this need, and to examine whether oral health can be promoted using a mobile app and a feature we have termed a “dental selfie.” In order to develop the app, we built a characterization protocol that included the following steps: (1) understanding user needs and perceptions toward the technology; (2) defining user identification and registration; (3) designing the UI/UX of the demo app screens; (4) creating user-administrator relationship chart and data retention systems; (5) connecting screens and user traffic on the app; (6) connecting screens and activities of the administrator.

As no apps of this nature are currently available, we went to dental clinics and observed dentist-patient interactions. Digital technology is widely used in dental clinics, and there is evidence that dentists appreciate the benefits of digital tools in their practices [47]. These tools enhance communication with peers and are perceived as useful in improving patient-dentist communication, management, and patient satisfaction [48,49]. After visiting the clinics, we conducted a focus group accompanied by semistructured, qualitative, in-depth interviews. Then we developed our first version of the app and tested it on 18 users. This pilot identified problems and enabled us to make the app more user-friendly. The app has 3 main characteristics: (1) It collects general information and information related to health behavior, with an emphasis on oral health; (2) it offers information about proper oral hygiene habits and the dangers of oral diseases, as well as visual tutorials demonstrating proper tooth brushing techniques; (3) dental selfie photographs are taken by the user and are evaluated by a dentist using the MGI.

The iGAM app was developed using the Agile approach, and the pilot study played an integral role in improving the features that users need. A randomized clinical trial in a large population and a qualitative study will follow and be reported separately.

### Limitations

The inclusion criterion for the pilot study was individuals aged 18 years and older; however, the ages of the volunteers all fell into the range of 18-45 years. This type of app may not be applicable for older people or those with poor literacy. This study included a diverse set of research methods and was developed in Hebrew because of the limits of self-funding. We recognize that the cultural diversity of individuals speaking other languages may correspond with different needs that the app will need to meet. Future research may address these limitations.

### Conclusions

The iGAM app is the first mHealth app for monitoring gingivitis to promote oral health using self-photography. iGAM presents a novel solution to improve the flow of information between dentists and their patients during the intervals between checkups. This app has tremendous potential for situations in which patients cannot meet their dentists in-person, such as during the COVID-19 pandemic. We believe that the collaboration between dentists, expert app developers, and typical dental patients—our target users—allowed us to make a quality and reliable app which will be updated regularly and improved.

## Abbreviations

COVID-19: coronavirus disease 2019
CSS: cascading style sheets
eHealth: electronic health
MGI: modified gingival index
mHealth: mobile health
TTS: text to speech
UI: user interface
UX: user experience
